# Phosphorylated Curdlan Gel/Polyvinyl Alcohol Electrospun Nanofibres Loaded with Clove Oil with Antibacterial Activity

**DOI:** 10.3390/gels8070439

**Published:** 2022-07-13

**Authors:** Dana M. Suflet, Irina Popescu, Irina M. Pelin, Geta David, Diana Serbezeanu, Cristina M. Rîmbu, Oana M. Daraba, Alin A. Enache, Maria Bercea

**Affiliations:** 1 Petru Poni Institute of Macromolecular Chemistry, Aleea Grigore Ghica Voda 41A, 700487 Iasi, Romania; ipopescu@icmpp.ro (I.P.); impelin@icmpp.ro (I.M.P.); diana.serbezeanu@icmpp.ro (D.S.); bercea@icmpp.ro (M.B.); 2 Department of Natural and Synthetic Polymers, Gh. Asachi Technical University, Bd. D. Mangeron 73, 700050 Iasi, Romania; dgeta54@yahoo.com; 3 Faculty of Veterinary Medicine, “Ion Ionescu de la Brad” University of Life Sciences, Aleea Mihail Sadoveanu 8, 700489 Iasi, Romania; crimbu@uaiasi.ro; 4 Faculty of Medical Dentistry, Apollonia University, Pacurari 11, 700511 Iasi, Romania; oana_daraba@yahoo.com; 5 ApelLaser S.A., Str. Vanatorilor 25, Ilfov, 077135 Mogosoaia, Romania; alin.enache@apellaser.ro

**Keywords:** curdlan phosphate, polyvinyl alcohol, clove essential oil, viscometer behaviour, rheological behaviour, electrospun, nanofibre membrane, antimicrobial test, viability cell test

## Abstract

Fibrous membranes based on natural polymers obtained by the electrospinning technique are a great choice for wound dressings. In order to promote an efficient wound repair, and to avoid antibiotics, antibacterial plant extracts can be incorporated. In the present work, the new electrospun nanofibre membranes based on monobasic phosphate curdlan (PCurd) and polyvinyl alcohol (PVA) were obtained for the first time. To establish the adequate mixing ratio for electrospinning, the behaviour of the PCurd and PVA mixture was studied by viscometry and rheology. In order to confer antimicrobial activity with the nanofibre membrane, clove essential oil (CEO) was incorporated into the electrospun solution. Well-defined and drop-free nanofibres with a diameter between 157 nm and 110 nm were obtained. The presence of CEO in the obtained nanofibres was confirmed by ATR–FTIR spectroscopy, by the phenolic and flavonoid contents, and by the antioxidant activity of the membranes. In physiological conditions, CEO was released from the membrane after 24 h. The in vivo antimicrobial tests showed a good inhibitory activity against *E. coli* and higher activity against *S. aureus*. Furthermore, the viability cell test showed the lack of cytotoxicity of the nanofibre membrane with and without CEO, confirming its potential use in wound treatment.

## 1. Introduction

Skin is the first defensive line of the body and has essential functions, such as protecting from microbial invasion, thermoregulation, tactile sensation, and contributing to immune responses to infectious agents by its several types of immune cells. Skin is permanently exposed to external factors and may lose its functionality due to chronic wounds, diabetic ulcers, burns, and acute injuries [1]. Microorganisms can quickly invade open wounds and grow rapidly, impeding the exudate role and delaying the wound’s healing [2,3]. Wound dressings are generally used to protect the wounds against infection or exposure to external aggressive factors. Currently, different types of wound dressings such as sponges, films, gels, and micro and nanofibres—from synthetic and natural polymers—are manufactured to protect the skin [4].

The nanofibre membranes are an ideal choice for wound dressings because of their functional versatility. They are able to mimic the fibrous architecture of the natural extracellular matrix (ECM) to support cell attachment and proliferation, are permeable to water vapour and oxygen, and can provide an effective barrier against bacteria or other microorganisms [1]. Electrospinning technology can be considered as a simple and low-cost procedure for generating micro and nanofibre membranes with some unique properties, including high surface-to-volume ratio, great mechanical properties, high porosity, and a homogeneous morphology compatible with ECM [1,5].

Polyvinyl alcohol (PVA) is a synthetic polymer, mostly used for electrospun nanofibres wound dressings by the electrospinning technique [6,7]. Even if it can be electrospun alone and used for wound dressings, the PVA has some disadvantages such as inappropriate hydrophobicity, inert bioactivity, and the inability to be used as bio-functional wound dressing for difficult healing wounds. These disadvantages have been removed by blending or co-electrospinning with natural polymers or other biomolecules leading to bioactive materials [8]. Natural biopolymers such as polysaccharides and their derivatives (alginates, chitosan, cellulose, curdlan, etc.), proteins (collagen, gelatine, fibrin, keratin) and proteoglycans are used for wound dressings preparation due to their biodegradability, biocompatibility, haemostatic activity, and non-toxicity [8,9,10]. We chose curdlan because it is a natural polysaccharide with a higher potential in biomaterials fabrication.

Curdlan is a bacterial polysaccharide resulting from the pure culture fermentation of *Agrobacterium biobar 1*, with a linear structure composed entirely of D-glucose units linked by β-(1–3) glucosidic bonds. Native curdlan was found to have immunomodulatory effects and anti-tumour activity [11,12,13,14] to increase the proliferation and migration of human keratinocytes by triggering dectin-1, and also to enhance the re-epithelialisation of ex vivo burn wounds [15]. However, its water insolubility is generally attributed to the triple-helical structure, and the extensive number of intra/intermolecular hydrogen bonds limits its applications; but, the introduction of ionic groups leads to the obtaining of water-soluble curdlan derivatives [16,17,18,19]. In spite of the biocompatibility and biological activity of curdlan and its derivatives, only few papers reported their use in electrospun membranes [20,21,22,23]. When simple curdlan was used together with PVA [16], PVA/chitosan [23], or poly (ethylene oxide) [22], toxic solvents, such as formic acid or NaOH solution, were used to solve the polysaccharide. When a water-soluble derivative of curdlan, carboxymethyl curdlan, was used in the electrospinning process by blending with PEO, the low viscosity of carboxymethyl curdlan (6 dL/g) led to the obtaining of non-uniform nanofibres with beads [21]. In addition, a mixture of water, 5% methanol, and 5% acetic acid was used for complete solubilisation of CM-Curd and PEO [21]. To remove any toxicity of the nanofibre membrane caused by the solvent, a totally soluble derivative with a high viscosity, such as monobasic phosphate curdlan (PCurd), can be used in the electrospinning process. PCurd has been used to obtain biocompatible materials [24,25]. To our knowledge, PCurd-based electrospun nanofibres have not been reported in the literature.

Generally, polymeric electrospun nanofibres do not present a satisfactory antimicrobial activity; therefore, novel wound dressings are developed by combing natural/synthetic polymers with antimicrobial and antioxidant agents, including traditional plant medicines with multifunctional activity to prevent infections, and facilitate wound healing [26]. Currently, there are many alcoholic/hydro-alcoholic extracts and essential oils extensively used in traditional wound treatment due to the phytochemical compounds, such as phenols, terpenoids, and alkaloids with antimicrobial, antioxidant, anti-inflammatory actions, etc. [26]. Clove essential oil (CEO) is an aromatic oil, extracted from the buds, leaves, or stems of *Eugenia caryophyllata*, and is a highly demanded product due to its traditional use in medicine, especially in dental health as an analgesic and antiseptic. The main component of CEO, eugenol, is mainly responsible for its special properties such as antioxidant, antibiotic, antimicrobial, analgesic, and anti-inflammatory properties [27,28]. It is reported that the total eugenol content in the oil extracted from clove buds can be around 36–90%, while in the oil obtained from leaves and stems it can increase to 75–90% and 85–95%, respectively [28]. Other major CEO compounds could be β-caryophyllene (13%) and eugenyl acetate, along with other compounds such as benzyl alcohol [28]. Eugenol has the disadvantage of instability in chemical and enzymatic degradation, as well as its losses due to volatilisation or thermal decomposition. The incorporation of natural therapeutic agents into a polymeric matrix can prevent their loss [29]. That is why nanofibres containing CEO that proved to have antimicrobial activity are proposed for wound dressings [30,31].

The aim of the present work is to obtain new PCurd/PVA electrospun nanofibre membranes that can be used as a wound protection dressing. Studies regarding the viscosity and rheological behaviour of the precursors, and of their mixture in solution, were performed in order to obtain information about the optimal mixture. In order to confer an antimicrobial barrier, the polymers mixture was loaded with CEO. The morphology of the new PCurd/PVA fibres, chemical composition (including total phenolic and flavonoids content), and the mechanical properties of the nanofibre membrane with and without CEO were evaluated. In addition, DPPH free-radical scavenging activity of the membrane and the in vitro CEO release behaviour were established. The cell viability and antimicrobial properties of CEO-loaded nanofibres were also analysed.

## 2. Materials and Methods

### 2.1. Materials

PCurd (with a degree of substitution up to 0.8, M_w_ ~270 kDa, determined by Gel Permeation Chromatography) was synthesised in our laboratory, using a method that was described elsewhere [17]. PVA (Mowiol^®^ 20–98, M_w_ = 125.000 g/mol, degree of polymerisation ~2800, degree of hydrolysis 98.0–98.8 mol%), potassium dihydrogen phosphate (KH_2_PO_4_), and disodium hydrogen phosphate (Na_2_HPO_4_) were purchased from Sigma-Aldrich, Chemie GmbH (Steinheim, Germany). The CEO (83.11% eugenol, 12.41% β-caryophyllene, 1.19% α-caryophyllene, etc) was purchased from BioSkin–Plafaria SRL (Iasi, Romania) and characterized by GC-MSD/FID gas-chromatography (Supplementary Data, Figure S1). Phosphate buffer solutions (PBS) with pH = 5.5 (KH_2_PO_4_ and Na_2_HPO_4_) were used for the in vitro release study. All the solutions were prepared with distilled water.

### 2.2. Methods

#### 2.2.1. Polymer Solution Preparation and Characterisation

The PCurd solution with 6% concentrations (*w*/*v*) was obtained by dissolving monobasic phosphate curdlan in distilled water at room temperature under magnetic stirring. The 10% (*w*/*v*) PVA solution was prepared by dissolving PVA in distilled water at 90 °C with stirring. The PCurd and PVA solutions were mixed in a 50:50 and 25:75 volumetric ratio and used in the electrospinning process. These PCurd/PVA solutions were diluted for the viscometry and rheology studies.

#### 2.2.2. Behaviour of PVA, PCurd and Their Mixture in Aqueous Solution

Viscometric measurements were performed with an Ubbehlode capillary viscometer for dilution series (type 0a, capillary diameter of 0.53 mm, and K constant of 0.005) at 25 ± 0.1 °C using an AVS 350 Schott automatic viscosity measuring system (Schott, Germany). Upon dilution, each polymer solution was kept about 15 min prior to the measurements for thermal equilibration. Flow times were obtained with good reproducibility: the errors were less than 1%. The shear flow curves of PCurd- and PVA-based solutions were obtained at 25 °C by using the MCR 302 Anton Paar rheometer (Graz, Austria) equipped with plate–plate geometry and Peltier device for temperature control. The viscosity was determined in steady shear conditions for shear rates ($\dot{\gamma }$) between 0.01 s^−1^ and 1000 s^−1^.

#### 2.2.3. Electrospinning Process of PCurd/PVA Solutions

The PCurd/PVA polymer solutions, with and without CEO, were electrospun using a Fluidnatek^®^ LE-50 laboratory line from Bioinicia S.L. (Valencia, Spain), equipped with a variable high voltage 0–30 kV power supply, at a voltage of 22.7 kV. The distance between the tip of the needle and the collector was adjusted to 14 cm, and the flow rate of the solution was set at 10 µL/min. The solution was fed from a 5 mL plastic syringe equipped with a 25 G stainless-steel needle (with an internal diameter of the needle equal to 0.5 mm) at the nozzle. The fibres were collected on a backer foil sheet attached to a copper grid used as collector. The electrospinning process was performed for 2 h at room temperature (25 ± 1 °C) and at a relative humidity of 48%. The electrospun membrane obtained from 6% PCurd and 10% PVA solutions were encoded as xPCurd/PVA (without CEO) or xPCurd/PVA/C_y_ (with CEO), where x is the volumetric percent of PCurd solution from the volume of the mixture, and y is the sample order number that depends on the amount of CEO in the mixture.

#### 2.2.4. Attenuated Total Reflectance-Fourier Transform Infrared Spectroscopy (ATR–FTIR)

The infrared spectra of the parent polymers and the electrospun nanofibre membrane with and without clove essential oil were recorded using a Bruker Vertex 70 (Billerica, MA, USA) spectrometer equipped with attenuated total reflection (ATR) with ZnSe crystal, in the wave number range from 4000 to 600 cm^−1^ at room temperature with a resolution of 2 cm^−1^.

#### 2.2.5. Scanning Electron Microscopy (SEM)

The surface morphology of PCurd/PVA electrospun nanofibres was investigated using a Verios G4 UC Scanning Electron Microscope (Thermo Scientific, Brno, Czech Republic). The samples were coated with 10 nm platinum, at 30 mA, using a Leica EM ACE200 Sputter coater to provide electrical conductivity and to prevent charge build-up during exposure to the electron beam. Image J software was used to determine the size and size distribution of the nanofibre diameter, considering at least 100 random points from the SEM images at 25k× magnification level.

#### 2.2.6. Mechanical Properties of the Electrospun Membranes

The mechanical testing was performed using a Texture Analyser (Brookfield Texture PRO CT3^®^, Brookfield Engineering Laboratories Inc., Middleborough, USA) at room temperature, following the ASTM D882 standard. The tensile tests were performed with rectangular samples (10 mm length, 6 mm width, and 0.06 mm thickness) with a trigger load of 0.067 N and a tensile rate of 0.1 mm/s. For better handling, the nanofibre membrane sample was placed between two paper frames and then it was attached to the clamps of the device [32]. Paper frames were cut before testing. The Young’s modulus *Y* was calculated from the slope of stress–strain curves between 0.5 and 1% elongation.

#### 2.2.7. Determination of Total CEO Content from the CEO-Loaded Electrospun Membrane

The total CEO content from CEO-loaded PCurd/PVA electrospun membrane was determined using the alcoholic extract method. Briefly, 1 cm^2^ of electrospun membrane (approx. 12 mg) was immersed in 4 mL of absolute ethanol and kept for 24 h in the dark under gentle shaking and room temperature, while ethanolic extract was obtained. Then, the absorbance was measured at 280 nm using a UV-Vis spectrophotometer (Thermo Fisher Scientific Evolution 201, Waltham, MA, USA). The total CEO content was calculated from the CEO calibration curve, determined using the ethanolic CEO solutions with various concentrations (5–80 μg/mL, y = 0.011x; R^2^ = 0.9972), and the results were expressed as μg CEO/ mg membrane.

#### 2.2.8. In Vitro CEO Release from PCurd/PVA Electrospun Membrane

The in vitro release studies of CEO from electrospun membrane were performed using a Franz diffusion cell, equipped with a Start-M^®^ membrane, a synthetic non-animal-based model for transdermal diffusion testing (Merck, Germany). The CEO-loaded electrospun membrane was placed on a Start-M^®^ membrane and the Franz cell was filled with buffer phosphate solution with pH 5.5 [33]. The release studies were performed at 32 °C, in order to simulate the physiological dermal conditions. At predetermined time intervals, the solution samples were withdrawn, and the CEO content was determined by spectrophotometric method at 280 nm. The same volume of the fresh buffer was added to replace the volume of the withdrawn samples. The amount of CEO released was determined using the calibration curve of CEO in the buffer solution (pH 5.5). Each experiment was conducted in triplicate.

#### 2.2.9. Phytochemical Proprieties of the CEO and CEO-Loaded Electrospun Membranes

##### DPPH Free-Radical Scavenging Activity

The antioxidant activity was evaluated spectrophotometrically through the free-radical scavenging capacity of the membrane using a 2,2-diphenyl-l-picrylhydrazil (DPPH) assay. The methodology described in the literature [34] was used with slight modifications in order to assess the DPPH free-radical scavenging capacity of CEO and CEO-loaded electrospun nanofibres. Briefly, 1 mL of ethanolic solution of CEO at different concentrations (1 × 10^−3^ to 0.1 μL CEO/mL) was mixed with 0.5 mL of ethanolic solution of DPPH (0.13 mm). The solutions were shaken and incubated for 30 min in darkness and at room temperature. Then, the absorbance was measured at 517 nm using a UV-Vis spectrophotometer. The DPPH scavenging effect was calculated as follows:

(1) $$DPPH^{\cdot }\;\left(\%\right)=\left(1-\frac{A_{s}}{A_{c}}\right)\times 100$$

where *A_c_* is the absorbance of the control sample (mixture of 1 mL ethanol with 0.5 mL DPPH^•^ solution), and *A_s_* is the absorbance of the tested sample. The low absorbance of the reaction mixture indicates high DPPH^•^ free-radical scavenging activity. IC_50_ which denotes the amount (μL) of CEO required to reduce the initial concentration of DPPH radicals by 50% was also calculated. Ascorbic acid was used as a standard.

The free-radical scavenging activity of CEO-loaded in electrospun membrane was determined. A total of 12 mg of each sample (approx. 1 cm^2^) was added to 1.5 mL DPPH ethanolic solution (0.13 mM). The solutions were shaken and incubated for various times (2, 4, 6, and 24 h) in darkness and at room temperature. The DPPH scavenging activity was determined using the method described above.

##### Determination of Total Phenolic Content (TPC)

The total phenolic content from commercial CEO and the CEO-loaded PCurd/PVA electrospun membrane was determined according to the Folin–Ciocalteu procedure [35]. Briefly, 1 mL of CEO ethanolic solution (0.1 μg/mL) was diluted with distilled water (9 mL) in a volumetric flask (25 mL). Folin–Ciocalteu’s reagent (0.5 mL, 2N) was added to the mixture and vortexed. After 5 min, 1.5 mL of Na_2_CO_3_ (20%) was added and incubated for two hours in the dark at room temperature. The absorbance was determined at 760 nm using a UV-Vis spectrophotometer. The TPC was calculated from the gallic acid (GA) calibration curve, and determined using the above method and the GA solutions with various concentrations (5–100 μg/mL). The results were expressed as mg GA/mL CEO. The TPC from the CEO-loaded electrospun membrane was established in the same manner using an ethanolic extract (see Section 2.2.7). Then, 1 mL of extract solution was used for determining the TPC using the method described above. The results were expressed as mg GA/mg membrane.

##### Total Flavonoid Content (TFC)

The total flavonoid content from commercial CEO and CEO-loaded PCurd/PVA electrospun membrane was estimated spectrophotometrically by the aluminium chloride method [35]. Briefly, 1 mL CEO ethanolic solution (0.1 μg/mL) or ethanolic extract (see Section 2.2.7) was diluted with distilled water (5 mL) in a volumetric flask (10 mL). Then, 0.3 mL NaNO_2_ 5% was added, and the flask was stored for 5 min in the dark at room temperature. Then, 0.6 mL AlCl_3_ 10% was added, and the mixture was again incubated for six minutes in the dark at room temperature. Then, 2 mL NaOH 1M was added and distilled water until the total volume reached 10 mL. The flask was kept again in the dark for ten minutes (until the colour changed from yellow to pale pink). The absorbance was read at 510 nm. The TFC was estimated using a Quercetin calibration curve (5–100 μg/mL) and the results were expressed as mg Q/mL CEO or mg Q/mg membrane.

#### 2.2.10. Antibacterial Assays

The antibacterial activity of PCurd/PVA with and without CEO was first determined using the Kirby–Bauer diffusimetric method. Standardised bacterial cultures *Staphylococcus aureus* ATCC 25923 (Gram-positive) and *Escherichia coli* ATCC 25922 (Gram-negative strain) were brought to a cell density corresponding to 0.5 McFarland turbidity standard (1.5 × 10^8^ CFU/mL). One mL of each bacterial suspensions was distributed into Petri dishes (90 mm) over which a Mueller-Hinton agar culture medium was placed; then, the PCurd/PVA samples with and without CEO (disk shape with a diameter of 7 mm and a weight of 5 mg) were placed. A gentamicin disk (10 µg) was used as a positive control. All Petri dishes were incubated at 37 °C for 24 h. The antimicrobial activity was assessed by measuring the diameter of the inhibition zones formed by each sample tested [36].

Antimicrobial activity was also tested using the ‘time-kill’ assay. The test was adapted for electrospun membranes to determine the antimicrobial efficacy over time (24 h and 48 h). This method compares the number of microorganisms in the initial suspension with the number of microorganisms still alive after contact with the test samples and at predetermined time intervals. Tests were performed with the same type of bacterial suspensions (*S. aureus* and *E. coli*) according to the standard 0.5 McFarland scale (1.5 × 10^8^ CFU/mL). Control tests were carried out on these suspensions, which consisted of distributing 1 mL of the suspension in Petri dishes with the culture medium (Mueller Hinton Agar), incubating at 37 °C, and examining the density of the microbial culture after 24 h (the cell density corresponded to 1.5 × 10^8^ CFU/mL). For the test samples, 5 mL of each bacterial suspensions were distributed in sterile tubes into which a PCurd/PVA membrane disc (7 mm diameter) with and without CEO was placed. The tested bacterial strains do not require special cultivation conditions, so after 24 h of incubation at 37 °C, 1 mL of bacterial suspension was taken from each tube and distributed into Petri dishes, and the same steps as for the anterior test were performed. This protocol was repeated after 48 h of incubation. After each incubation time interval, the bacterial colonies formed in the culture medium were counted and expressed as colony-forming units (CFU/mL). The working diagram is shown in Supplementary Data, Figure S2.

Antimicrobial efficacy was defined by calculating the difference between the number of living cells in the initial suspension (1.5 × 10^8^ CFU/mL) and the number of bacterial cells that survived after the test. All values obtained were converted to the log_10_ scale, whereupon the log reduction (LR) and the percentage of log reduction (LR%) were calculated according to the formulas:

Log_10_ reduction (LR) = mean log_10_ (microbial population) − mean log_10_ (surviving population) (2)

Percent Reduction (%) = 100 × (1 − 10^-LR^) (3)

The higher the log reduction, the better the antibacterial efficacy of the product. For example, a reduction of 1 log corresponds to a reduction of 90% of the bacteria. The bactericidal potential is highlighted at a reduction of 3 log_10_ CFU/mL, which corresponds to the destruction of 99.9% of the bacteria in the inoculum [37].

#### 2.2.11. Cell Viability Assay of Electrospun Nanofibres Membrane

The cytotoxicity analysis of the electrospun nanofibres membrane was performed using Human Dermal Fibroblasts adult. The cells were grown in DMEM (Dulbecco’s Modified Eagle Medium), supplemented with 10% FBS (Fetal Bovine Serum) and an antibiotic cocktail consisting of 1% (*v*/*v*) penicillin-streptomycin and 1% (*v*/*v*) non-essential amino acids. The medium was changed every day. The cell culture flasks (NuncTM EasYFlask TM, ThermoFisher Scientific, USA) were incubated at 37 °C in a 95% humidified atmosphere and 5% CO_2_ (MCO-5AC CO_2_ Incubator, Sanyo). Cells were allowed to grow in four culture flasks to reach 80% confluence and then were trypsinised with 0.25% trypsin solution at 37 °C for 3 min, followed by the addition of fresh medium to neutralize trypsin. The concentration of cells was 1 × 10^4^ cells/cm^2^. After centrifugation (Roto-fix-32A, Hettich, Tuttlingen, Germany) and re-suspension in fresh medium, the viable cells were incubated in the presence of sterilised membrane samples on flat-bottom 96-well plates for 24 and 48 h. After the incubation time, the cell viability was determined using 3-(4,5-Dimethyl-2-thia zolyl)-2,5-diphenyl-2H-tetrazolium bromide (Merck, Darmstadt, Germany) following the MTT assay. Each type of nanofibre membrane was tested in triplicate.

#### 2.2.12. Statistical Analysis

Data were presented as mean ± SD. The ANOVA Single Factor was performed for statistical analysis and *p* < 0.05 was considered to be statistically significant.

## 3. Results and Discussions

### 3.1. Behaviour of PVA, PCurd and Their Mixture in Aqueous Solution

The processability of the polymeric solution and the morphology of the obtained fibres are correlates with the rheology of the polymeric solution [38]. When composite nanofibres are envisaged, the miscibility between the components, which can be studied by viscometry and rheology, is also very important. 

**Figure 1:**
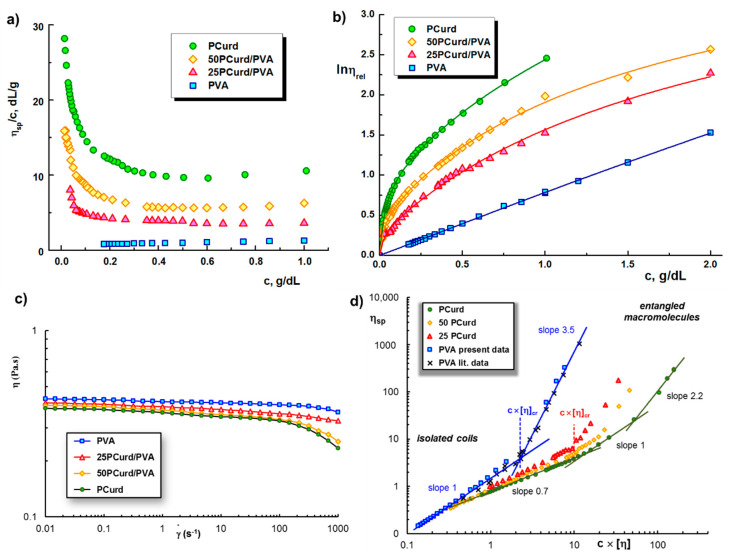
(**a**) Dependence of reduced viscosity and (**b**) natural logarithm of the relative viscosity as function of polymer concentration for PCurd, PVA, and PCurd/PVA mixture in pure water at 25 °C; (**c**) Shear viscosity as a function of shear rate for PVA, PCurd, and PVA/PCurd mixtures at 25 °C; (**d**) Specific viscosity as a function of *c* × [*η*] for PVA, PCurd, and PVA/PCurd mixtures in aqueous solution at 25 °C. Other reported data for PVA were included [46].

The hydrodynamic properties of the PCurd/PVA mixture solutions and their components (PCurd and PVA) were studied by capillary viscometry over a large concentration range (0.005 ÷ 2 g/dL). The Huggins plots for precursor solutions in pure water at 25 °C are presented in Figure 1a. Typical polyelectrolyte behaviour of the solutions containing PCurd is observed: the reduced viscosity increases exponentially with decreasing polymer concentrations, due to the uncoiling of the charged chains in the dilute medium [17,19]. 

In order to obtain the intrinsic viscosity of polyelectrolytes and of the binary mixtures of polyelectrolyte/neutral polymer [39,40,41] in aqueous solution without added salt, the non-linear Wolf equation can be applied [19,42,43,44]. According to the Wolf approach, the intrinsic viscosity can be determined from the initial slope of dependence between the natural logarithm of relative viscosity (*ln η_r_*) and polymer concentration (*c*) according to the following Equation:

(4) $$\ln\eta_{r}=\frac{\tilde{c}+\propto {\tilde{c}}^{2}}{1+\beta \tilde{c}+\gamma {\tilde{c}}^{2}}$$

where $\tilde{c}$, called the reduced polymer concentration, is given by:

(5) $$\tilde{c}=c\times \left[\eta \right]$$

where [*η*] is the intrinsic viscosity and *α*, *β*, and *γ* are system specific parameters. The parameter *α* gives the friction between the components as a function of composition, while *β* and *γ* consider the changes of free volume in polymer solutions as compared with the pure solvent. The dimensionless parameter *c* × [*η*] provides an index of the total volume occupied by the polymer. It was proven that Equation (5) gives the concentration dependence of any macromolecule in solution over the entire range of concentration [40,41,45]. Figure 1b shows the dependence of the viscosity vs. concentration (according to Equation (4)) for PCurd, PVA, and their selected mixtures in aqueous solution at 25 °C. The lines are obtained by means of Equation (4), by considering *α* = 0 and the values of parameters *β* and *γ* listed in Table 1. The high value of [*η*] for PCurd is due to the rigidity of the polysaccharide chains that are in extended conformation [17]. This value has a significant decrease with the increase ratio of PVA in the mixture.

Figure 1c presents the shear flow curves of the investigated systems at 25 °C. The Newtonian behaviour is observed at low shear rates and the viscosity values are closed for PVA and PCurd, facilitating the homogenisation and stability of the PVA/PCurd mixtures. The viscosity starts to decrease at high shear rates ($\dot{\gamma }$ > 100) and non-Newtonian behaviour appears for PCurd-based solutions at lower shear rates, suggesting higher relaxation times for polysaccharide solutions. The viscosity and rheology data allow the delimitation between entangled and non-entangled states of polymers in the solution. Figure 1d shows the dependence of the specific viscosity for polymers in aqueous solutions at 25 °C, as a function of the dimensionless parameter *c* × [*η*]. For *c* ≤ 1 g/dL (PCurd solutions) or 2 g/dL (PVA containing systems), the $\eta_{sp}$ values were determined by capillary viscometry, while for *c* > 1 g/dL, $\eta_{sp}$ was obtained from shear flow measurements at 25 °C (for the solvent, the value of 0.89 × 10^−3^ Pa·s was considered). For PVA solutions, two linear dependences are evidenced in the plot of $\eta_{sp}$ vs. *c* × [*η*], delimiting the critical value (*c* × [*η*])*_cr_* = 2.1, from which the entangled concentration, *c_entangled_*, can be estimated (see Table 1). This critical concentration delimits two different states: the isolated coils (non-entangled state) where *η_sp_* scales as (*c* × [*η*])^1^, and *network solution regime* (entangled state) for which *η*_sp_∞ (*c* × [*η*])^3.5^, and the exponent 3.5 is in agreement with previous data on PVA solutions [46]. In the presence of polysaccharide chains, *c_entngled_* decreases (Table 1). Above the critical concentration, *c*_*entangled*_ and the macromolecular coils touch one another, and entanglements start to appear. In order to ensure the continuity of the fibres, entangled polymer solutions with a concentration of about 2.5 × *c_entangled_* are recommended for the electrospinning process [47,48]. The fibre diameter depends on the solution concentration [49]. Based on the dependences shown in Figure 1d, the PCurd/PVA mixture with a 25:75 volume ratio (25PCurd/PVA) was selected for preparing the composite fibres, because the polymers in this composition are in an entangled state at *c* × [*η*] ≈ 10. This sample preserves the high stability of PVA in continuous shear conditions (Figure 1c).

### 3.2. Membrane Characterisation

**Figure 2:**
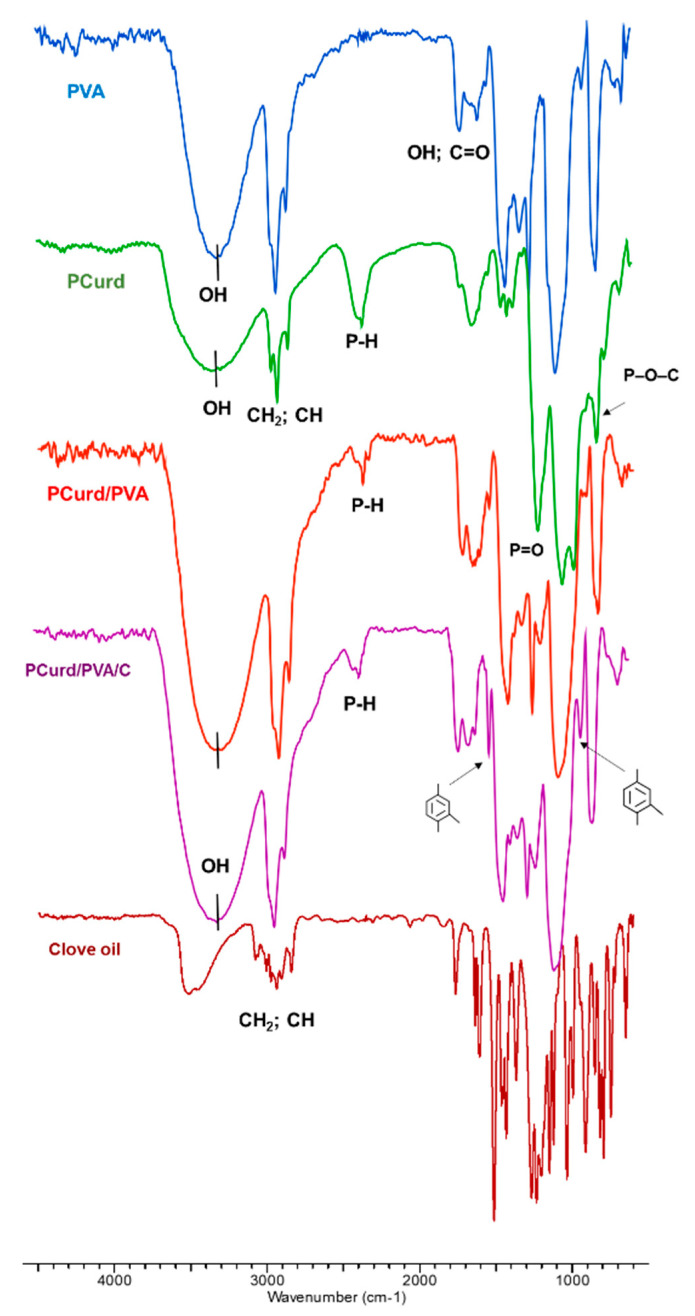
The ATR spectra of the nanofibre membranes with and without incorporated CEO (PCurd/PVA, PCurd/PVA/C) together with the polymeric components and the therapeutic oil.

The chemical structure of the PCurd/PVA nanofibres membrane was confirmed by ATR spectrometry (Figure 2). The following characteristic bands of the partners (PCurd and PVA) were observed in the ATR spectrum of the PCurd/PVA electrospun membrane: a large band at 3454–3295 cm^−1^ corresponding to the OH groups; a band at 2939–2855 cm^−1^ attributed to the CH_2_ and CH groups; a band at 1715 cm^−1^ related to the stretching vibrations of –OH groups and C=O, which can be ascribed to the acetate groups of PVA molecules; the band at 1543–1643 cm^−1^ characteristic of C–OH from the glucosidic units of polysaccharide and polyvinyl chains; the bands between 1458–1325 cm^−1^ corresponding to CH_2_ bonds; the bands from 2367 cm^−1^ (P–H), 1211 cm^−1^ (P=O), 978 cm^−1^ (P–OH), and 827 cm^−1^ (P–O–C bond), corresponding to the monobasic phosphate curdlan derivate [17,50].

In the spectrum of CEO, the signals in the wavenumber range of 746–1267 cm^−1^ were attributed to the C=C bonds from the side chains and from the aromatic rings of eugenol or eugenyl acetate [51]. Moreover, the sharp peaks at 1637, 1607, and 1512 cm^−1^ are attributed to the C=C stretching vibration. The peak at 1267 cm^−1^ can be ascribed to the characteristic bands of =C–H in-plane bending absorption of the aromatic rings and –CH_2_ swing in alkanes; meanwhile, the peak at 1232 cm^−1^ is likely due to the C–O–C symmetric expansion of aromatic acid esters and C–OH vibrational stretching of phenolic compounds. The peaks from 1150 cm^−1^ and 1034 cm^−1^ can be ascribed to the stretching vibrations of C–O and the deformation vibration of C–OH groups. Likewise, the vibration absorption of RHC=CHR from β-caryophyllene can be observed at the wavenumber of 648 cm^−1^ [52,53].

In the spectrum of CEO-loaded nanofibres (PCurd/PVA/C), the bands at 1514 and 914 cm^−1^ attributed to the aromatic rings from the CEO compounds were observed, confirming the inclusion of the therapeutic oil in the electrospun membrane. The other bands’ characteristics for CEO from the PCurd/PVA/C membrane are overlapped with the bands of the polymeric matrix.

**Figure 3:**
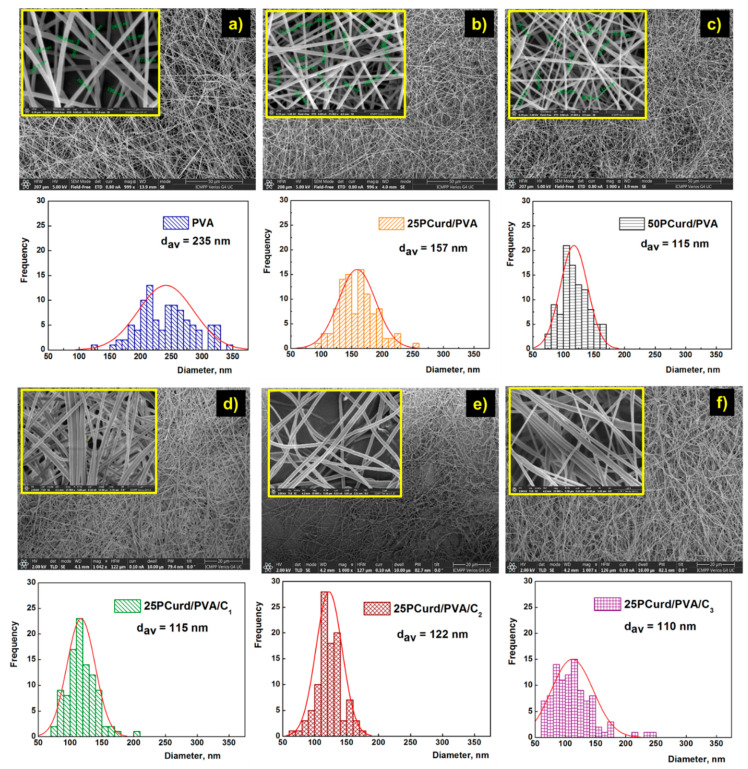
Scanning electron micrographs of the electrospun nanofibre membrane with and without clove essential oil (general view with detail and size distribution): PVA (**a**), 25PCurd/PVA (**b**), 50PCurd/PVA (**c**), 25PCurd/PVA/C_1_ (**d**), 25PCurd/PVA/C_2_ (**e**), and 25PCurd/PVA/C_3_ (**f**) membranes.

SEM micrographs of the electrospun membranes obtained from PVA and PCurd/PVA mixtures show the drop-free nanofibres (Figure 3a–c). The average diameter of nanofibres decreases from 235 nm to 115 nm with an increasing PCurd content in the polymer mixture (Table 2). It is known that polyelectrolytes induce through their ions an increase in the charge carrying capacity of the polymer jet due to the increase of electrical conductivity in the mixture [54]. The polymer jet is subjected to increased tension in the electric field during the electrospinning process, and the high conductivity of the solution determines an extended elongation and the obtaining of fibres with a smaller diameter [54,55,56]. The presence of CEO in the membranes (25PCurd/PVA/C) also led to uniform nanofibres without beads (Figure 3d–f) but with a lower average diameter (110–120 nm) than the nanofibres without CEO (157 nm). The CEO content did not significantly influence the average diameter of the nanofibres, but did influence the size distribution. However, the thickness of fibres slightly increases from 115 nm (25PCurd/PVA/C_1_) to 122 nm (25PCurd/PVA/C_2_) with an increase in the CEO content, probably due to a decrease the electrical conductivity of the solution and an increase in the solution viscosity as an effect of some interactions (such as hydrogen bonds) between the CEO and polymer chains. The same behaviour was also reported in the case of CEO-loaded PEO nanofibres [57]. Nevertheless, it can be said that uniform nanofibres can be obtained using PVA and PCurd, even with a high CEO content.

### 3.3. Mechanical Properties

**Figure 4:**
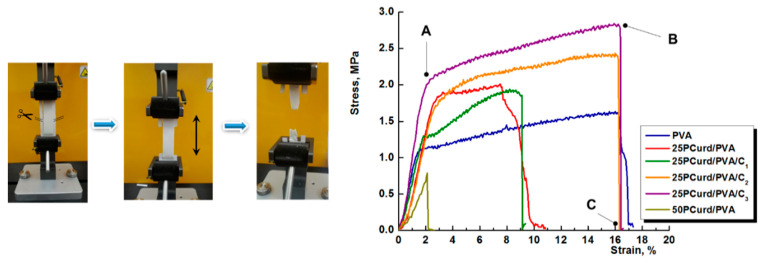
Stress–strain tensile curves for PVA and PCurd/PVA nanofibre membranes with and without with CEO.

The investigation of the mechanical properties is a key-test for electrospun membranes. The mechanical behaviour of the developed nanofibre membranes is shown in Figure 4 and the main properties (the elastic modulus, ultimate tensile strength, elongation at break) are listed in Table 2. The tensile strength of the nanofibre membranes depends on several factors, such as fibre strength, fibre diameter, fibre surface morphology, fibre orientation in membrane structure, and frictional forces between the fibres [58,59]. As is shown in the Figure 4, the stress–strain curves of the membranes present three distinguished zones: the first zone 0-A starts with the fibre straightening and reorienting towards the direction of the applied load without fibre breakage, followed by fibre breakage and slippage till it reaches the maximum stress (A-B zone), and finally the residual zone when the fibres slip until the final failure of the whole sample (B-C zone) [58].

The presence of PCurd in the electrospun nanofibre membrane led to the decrease in the second zone (A-B) until its absence for 50PCurd/PVA, probably due to a decrease in fibre diameter and a reduction in the contact area between the fibres that lead to a decrease in frictional force. That is why the elongation at break decreases from PVA membrane (16.08%) to 25PCurd/PVA (7.58%) and 50PCurd/PVA (2%).

The first zone (0-A) where the fibres stretched and reoriented is larger for the 25PCurd/PVA sample, showing that PCurd increased the elasticity and the tensile strength of the fibres. The Young’s modulus of the PVA and 25PCurd/PVA membranes are relatively closed, at 0.924 MPa and 0.861 MPa, respectively. A high percentage of PCurd (37%) in the electrospun mixture led to the decrease in all the mechanical properties of the electrospun membrane; but, 17% PCurd in the mixture improves the tensile strength of the membrane. These results, together with the rheological studies, led to the choice of 25Pcurd/PVA sample for the incorporation of CEO.

The Incorporation of a small amount of CEO (2.70%) in the electrospun membrane (25pCurd/PVA/C_1_) induced a slight, not significant decrease in Young’s modulus and the ultimate tensile strength, and an increase in elongation at break compared to the 25PCurd/PVA, showing that CEO acts as a plasticiser. Increasing the CEO content to 5.26 and 35.7% led to an increase in tensile strength to 1.97 MPa and 2.77 MPa, respectively. The elongation at break and Young’s modulus also increase with increasing the CEO content. This mechanical behaviour of the loaded PCurd/PVA electrospun membrane could be attributed to the intermolecular interactions between the polymers chains and the low molecular compound (CEO). These new interactions led to the increase in friction force between the fibres and, therefore, an increase in ultimate tensile strength and the elongation at break without the change of Young’s modulus magnitude [60,61].

### 3.4. In Vitro CEO Release

**Figure 5:**
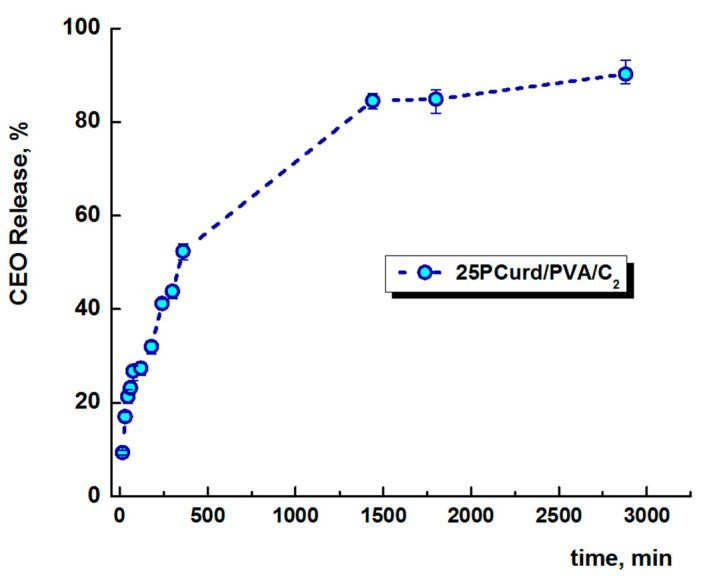
The release profile of clove essential oil from 25PCurd/PVA/C electrospun membrane at 32 °C and pH 5.5.

The loaded clove oil release rate from electrospun nanofibres is shown in Figure 5. The in vitro release studies of CEO from the electrospun membrane were performed in the physiological dermal conditions (pH 5.5 and 32 °C) using a Franz diffusion cell, equipped with a Start-M^®^ membrane, as a model for transdermal diffusion testing. The release profile shows a faster release in the first 8 h (52%), followed by a slow and continuous release for the subsequent 48 h. A total of 85% of the CEO was released after 24 h. The retarded release of CEO from PCurd/PVA/C nanofibres is an advantage of this electrospun membrane to be used as a wound protection dressing. The same results are reported for CEO release encapsulated in chitosan and poly-ethylene oxide nanofibres [57].

### 3.5. Phytochemical Proprieties of the CEO-Loaded Electrospun Membranes

The CEO content from the 25PCurd/PVA/C electrospun fibres was determined, and the values are presented in the Table 2. The values are close to the theoretical CEO content, with the exception of the 25PCurd/PVA/C_3_ sample where the CEO content was lower (27% compared with 35%), due to the evaporation of the volatile contents.

DPPH and total phenolic and flavonoid content tests are valuable tools for assessing the biological activity of natural products (essential oils) loaded in membranes/films used in the medical and pharmaceutical fields. Antioxidant activity is indicated to have a positive effect on wound healing by minimising reactive oxygen species generated by senescent cells and lipid peroxidation [62]. The CEO’s encapsulation process was aimed to enrich the electrospun membrane with new properties such as antioxidant and antimicrobial activity for future applications as a wound protection dressing. 

**Figure 6:**
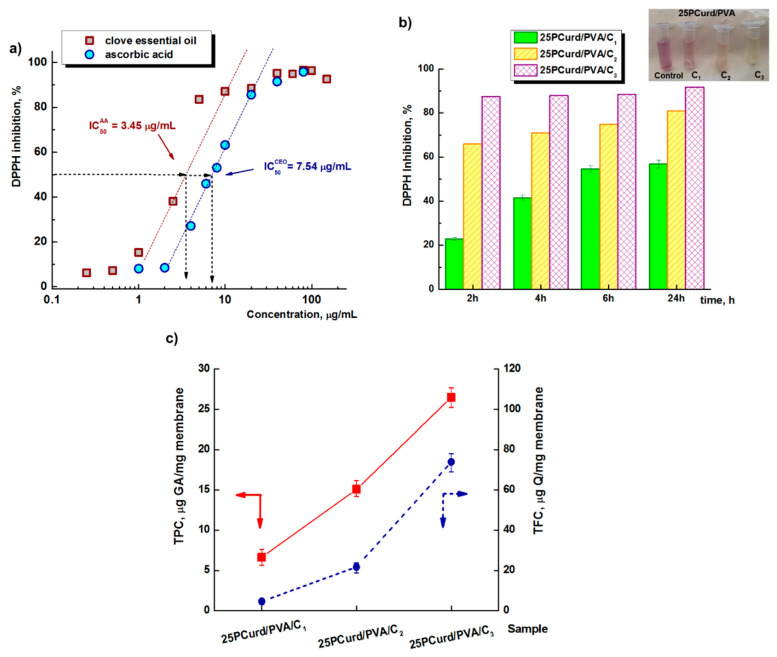
DPPH free-radical scavenging activity at different concentrations of CEO and ascorbic acid as reference (**a**), the CEO-loaded 25PCurd/PVA electrospun membrane (**b**), and the total phenolic content (TPC) and total flavonoid content (TFC) from CEO-loaded 25PCurd/PVA electrospun membrane (**c**).

The DPPH assay highlights the free-radical scavenging properties of CEO loaded in 25PCurd/PVA. Hydrogen atom- and electron-donating antioxidants convert the DPPH^•^ radical (violet) into its non-radical form (DPPH-H, yellow) causing a decrease in its characteristic absorbance at 515 nm. Figure 6a shows the scavenging activity of CEO on DPPH^•^ radicals at various concentrations compared with ascorbic acid activity. The 95.84% maximum inhibitory activity of CEO was observed at 0.1 μg/mL, comparable with the literature data that found a maximum inhibitory activity of clove-leaf essential oil of 91.2% at 0.5 μg/mL [63,64,65]. The IC_50_ (50% inhibition of the DPPH free radical) of CEO was calculated as 7.54 μg/mL. The positive control used in this research was ascorbic acid for which a free-radical scavenging activity of DPPH of 96.47% and IC_50_ of 3.45 μg/mL was established (Figure 6a).

Figure 6b shows the DPPH free-radical scavenging activity of the CEO-loaded 25PCurd/PVA electrospun membrane. A gradual increase from 22.8% inhibition, after 2 h immersion in DPPH solution to 57% after 24 h was observed for the membrane with the lowest CEO content (2.70%), while a CEO content of 5.26% in the PCurd/PVA mixture led to a relatively sharp increase of over 65% inhibition in the first 2 h of contact and 80% after 24 h. The maximum DPPH free-radical scavenging activity (87–92%) was observed for the 25PCurd/PVA/C_3_ membrane from the first hours of contact, a value very close to that of pure CEO (95.84%).

The *total phenolic* (TPC) and *flavonoid content* (TFC) from CEO and from the 25PCurd/PVA/C membrane were calculated using the Gallic acid and Quercetin calibration curves, respectively. The phenolic acids (eugenol, allylphenol) are found in high concentration in CEO leading to TPC = 387 μg GA/mg CEO, while the flavonoid compounds, such as kaempferol, quercetin, and glycosylated derivatives, are also present in clove but in a lower amount: TFC = 405 μg Q/mg CEO. These values are in accordance with the literature [63]. The presence of CEO in the 25PCurd/PVA/C nanofibre membrane was also demonstrated by determining the TPC and TFC of the loaded membranes (Figure 6c). As expected, TPC and TFC increase with increasing clove oil content in the electrospun fibres. In the case of 25PCurd/PVA/C_2_ the phenol content and the flavonoid content was 15 μg GA/mg membrane and 21.7 μg Q/mg membrane, values which are comparable with the theoretical values obtained from the CEO content of the membrane (Table 2). 

### 3.6. Antimicrobial Test

The antibacterial activity of the PCurd/PVA electrospun membrane with and without CEO was evaluated using *S. aureus* (Gram-positive strain) and *E. coli* (Gram-negative strain) bacteria. Antimicrobial activity was assessed by measuring the diameter of the inhibition zones and comparing with the positive control (gentamicin 10 μg). The results of the triplicate test are presented in the form of the arithmetic mean in Table 3. All PCurd/PVA samples with CEO showed antimicrobial activity, but in different proportions depending on the composition of the bioactive compounds and the strain of bacteria against which the test was performed. PCurd and PVA do not present the antimicrobial activity (Figure S3 and Table S1 from Supplementary Data), so this effect is due to the loaded CEO. As in other studies [36,66], the different sensitivity of Gram-positive and Gram-negative bacterial strains to the action of natural antimicrobial agents can be observed. This behaviour can be attributed to the structure of the bacterial cell wall, which contains a more diffusible mucopeptide layer in the case of Gram-positive bacteria, while the in the case of Gram-negative bacteria, the diffusion rate of lipophilic antibacterial compounds was reduced by a lipopolysaccharide layer [52,67]. So, the 25PCurd/PVA/C_3_ sample shows a higher value of the average diameter of the inhibition (8.97 mm) for *S. aureus* compared with *E. coli* (8.10 mm). 

The antimicrobial action of CEO is attributed to eugenol, oleic acids, and lipids contained in the essential oils. The mechanism of action of essential oils depends on their chemical composition, and their antimicrobial activity is not attributed to a single mechanism but is a cascade of reactions involving the entire bacterial cell [68]. However, it is known that antimicrobial activity depends on the lipophilic nature of the components. The components penetrate the cell membranes and mitochondria of microorganisms and, among other effects, inhibit the flux of electrons bound to the membrane and energy metabolism. This leads to a breakdown of the proton pump and depletion of the ATP tank. High concentrations can also lead to lysis of cell membranes and denaturation of cytoplasmic proteins [68,69,70]. In addition, clove essential oil could exert an inhibitory effect during the processes of transcription of DNA strands into mRNA, the translation of mRNA into protein, and DNA replication [70].

The antimicrobial activity spectrum of the positive control (gentamicin 10 μg) obtained by testing the *S. aureus* (24 mm) and *E. coli* (22 mm) strains was in accordance with the specific standards [71]. The antimicrobial activity of the CEO-loaded PCurd/PVA nanofibre membrane can be considered less effective than gentamicin (control), but the toxicity of synthetic antibiotics and induced antimicrobial resistance, which is currently considered a major global threat to health and development, must be taken into account [72,73]. It is well known that plants are an almost inexhaustible source of antimicrobial compounds (flavonoids, alkaloids, tannins, terpenoids, etc.) that act simultaneously and through complex mechanisms, with a minimal likelihood of microbial resistance development [74].

### 3.7. Time Kill Assay Test

In vitro antimicrobial efficacy in suspension is more sensitive compared with the disc diffusion test. In this method, the difference between the number of bacterial cells initially in the microbial suspension (1.5 × 10^8^ CFU/mL equivalent to 8.176 Log_10_ CFU/mL) and the number of bacterial cells that survived in the suspension after 24 and 48 h of contact with the sample is converted to the logarithmic scale (log_10_). Logarithmic reduction (LR) and the percentage of logarithmic reduction (LR%) were used to interpret the results of tested 25PCurd/PVA electrospun membranes with and without CEO (Table 4). 

**Figure 7:**
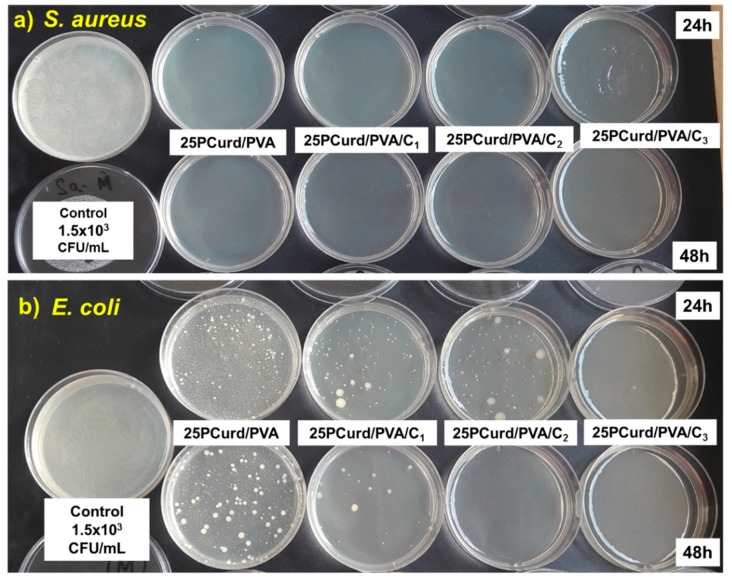
Time-kill assay test of PCurd/PVA samples with and without CEO against *S. aureus* (**a**) and *E. coli* (**b**).

All 25PCurd/PVA samples with and without CEO tested on *S. aureus* induced antimicrobial activity after the first 24 h, which persisted up to 48 h of incubation and reached a log reduction of 8176 log10 CFU/mL, corresponding to 100% efficiency (Table 4, Figure 7).

In the case of *E. coli,* the analysis of antimicrobial efficiency showed a very good antimicrobial activity over time, as shown by the percentage of log reduction between 99.92% and 100%. Thus, when testing the sample 25PCurd/PVA, the lowest log reduction of 4.809 log_10_ CFU/mL was achieved after only 24 h, and 5.109 log_10_ UFC/mL after 48 h of incubation. The presence of CEO enhanced the antimicrobial activity so that after 24 h, the reduction against *E. coli* was 6.213 log_10_ UFC/mL, and 6.834 log_10_ UFC/mL after 48 h for the 25PCurd/PVA/C_1_ sample. The increased concentration of CEO further affected the antimicrobial activity from 6.257 CFU/mL to 8.176 CFU/mL for 25 PCurd/PVA/C_2_ and a maximum log reduction of 8.176 log_10_ CFU mL (100% antimicrobial efficacy) for 25 PCurd/PVA/C_3_. The antimicrobial activity against *E. coli* was also lower compared to *S. aureus*, in accordance with the diffusion tests. 

### 3.8. Cytotoxicity Assay

**Figure 8:**
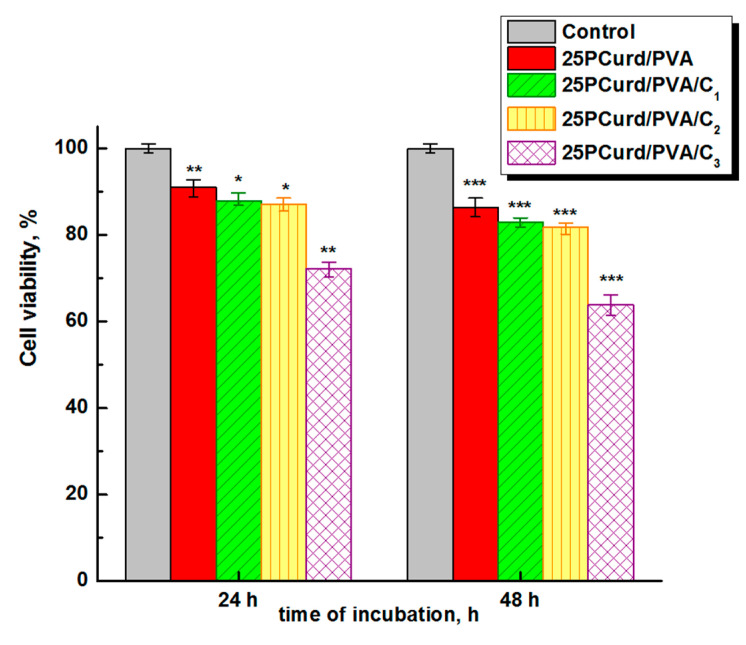
Cells viability of nanofibre 25PCurd/PVA with and without cove oil after 24 and 48 h (* *p* < 0.001, ** *p* < 0.01, *** *p* < 0.05).

Biocompatibility and the lack of cytotoxicity are imperative requirements when designing new wound dressing materials, but generally, it is a contradiction between antimicrobial activity and cell viability [75,76]. It is known that CEO can have a toxic potential not only for bacteria, but also for living and healthy cells, above a certain concentration [77]. Taking into account these aspects, the viability of dermal fibroblasts in direct contact with nanofibre membranes with and without CEO was investigated. The results showed a slight decrease in cell viability with an increase in CEO content, from 91% (after 24 h) and 86.38% (after 48 h) for 25PCurd/PVA without CEO to 72.26% (after 24 h) and 64.0% (after 48 h) for samples with the highest CEO content (Figure 8). Even if the cell viability was relatively low (64.0%), these results place the 25PCurd/PVA/C_3_ sample on the threshold of cytotoxicity and non-cytotoxicity. From the antimicrobial tests and the cytotoxicity assay it can be asserted that 25PCurd/PVA/C_2_ is non-toxic and also has a good antimicrobial activity. 

## 4. Conclusions

New nanofibre membranes based on PCurd and PVA were obtained by the electrospinning process from aqueous solution. Rheological and viscometric studies of PCurd/PVA mixture in solution suggest that a volumetric ratio of 25:75 between 6% PCurd and 10% PVA solutions, corresponding to a gravimetric ratio between the polymers of 17:83, is appropriate for the electrospinning. The tensile tests of the fibrous membrane also showed that good mechanical properties (tensile strength of 1.97 MPa, Young’s modulus of 0.86 MPa) are obtained at this composition. CEO can be incorporated in high amounts (until 35%) in the PCurd/PVA electrospun membranes with the obtaining of well-defined uniform nanofibres, without drops and with diameters between 60 and 180 nm. The tensile tests shown that CEO acts as plasticiser leading to the increase in membrane elongation. The incorporation of the therapeutic agent was demonstrated by ATR–FTIR, and by the measurements of the TFC and TPC of the electrospun nanofibres. These membranes have antioxidant properties, with the DPPH inhibition activity being influenced by the amount of incorporated CEO and by the contact time. In physiological conditions of the skin, 83% of CEO was released from the electrospun membrane in 24 h. The antimicrobial activity studies using the disc-diffusion method showed that PCurd/PVA/C samples have good inhibitory activity against *E. coli* and higher activity against *S. aureus*. The modified time-kill assay proved that, in suspension, the membranes reduces 100% of the development of *S. aureus*, regardless of the CEO content. In the case of *E. coli*, the reduction is between 99.92% and 100%, being influenced by the content of the antimicrobial agent. The cell viability assay showed that the nanofibres with and without small amounts of CEO are non-cytotoxic. The sample with a high amount of essential oil (27%) is on the threshold of cytotoxicity and non-cytotoxicity. It can be said that 5.6% CEO is enough for a high antimicrobial activity of the membrane without toxicity. These results encourage studies regarding the use of these materials as a wound protection dressings.

## Figures and Tables

**Table 1:** The viscometric parameters determined for aqueous solutions of PCurd, PVA, and their mixtures at 25 °C, according to Wolf model.

Sample Code	PCurd/PVA Ratio, (*v*/*v*)	[*η*] (dL/g)	β	γ	*c_entangled_* (g/dL)
PCurd	100/0	26.032 ± 0.8707	0.6338 ± 0.0117	−0.0123 ± 0.0004	<1.9975
50PCurd/PVA	50/50	9.808 ± 0.6121	0.5917 ± 0.0241	−0.0135 ± 0.0010	2.0392
25PCurd/PVA	25/75	5.227 ± 0.2897	0.6297 ± 0.0306	−0.0279 ± 0.0019	1.9133
PVA	0/100	0.792 ± 0.0132	0.0750 ± 0.0036	0.0109 ± 0.0018	2.6509

**Table 2:** The composition and the main characteristics in terms of composition of the PCurd/PVA nanofibre membranes.

Sample Code	Electrospinning Composition			d_av_ (nm)	μg CEO/ mg Membrane	Mechanical Properties		
	PCurd/PVA Ratio, (*v*/*v*)	PCurd Content, (%, *w*/*w*)	CEO (%, *w*/*w*)			σ (MPa)	ε_b_ (%)	Young’s Modulus (MPa)
PVA	100/0	0	-	235 ± 7.37	-	1.63 ± 0.17	16.08 ± 1.85	0.924 ± 0.19
25PCurd/PVA	25/75	17	-	157 ± 3.09	-	1.97 ± 0.16	7.58 ± 0.90	0.861 ± 0.13
50PCurd/PVA	50/50	37	-	115 ± 3.10	-	0.788 ± 0.08	2.01 ± 0.45	0.392 ± 0.05
25PCurd/PVA/C_1_	25/75	17	2.70	115 ± 1.36	2.88	1.93 ± 0.14	8.73 ± 0.55	0.831 ± 0.12
25PCurd/PVA/C_2_	27/75	17	5.26	122 ± 2.07	5.63	2.40 ± 0.21	16.13 ± 1.8	0.861 ± 0.10
25PCurd/PVA/C_3_	27/75	17	35.71	110 ± 1.47	27.12	2.77 ± 0.25	16.38 ± 2.5	1.309 ± 0.50

**Table 3:** The average diameter of zone of inhibition bacterial growth (samples diameter 7 mm).

Sample Code	μg CEO/Disc	*S. Aureus* (mm)	*E. coli* (mm)
25PCurd/PVA	-	7.00	7.00
25PCurd/PVA/C_1_	10.35	7.93	7.10
25 PCurd/PVA/C_2_	57.49	8.12	7.30
25PCurd/PVA/C_3_	162.71	8.97	8.10
Gentamicin (10 μg)	-	24	22

**Table 4:** Bacterial logarithmic reduction after 24 h and 48 h of contact with 25PCurd/PVA electrospun membrane with and without CEO.

Bacteria Strains		M(+)	Samples							
			25PCurd/PVA		25PCurd/PVA/C_1_		25PCurd/PVA/C_2_		25PCurd/PVA/C_3_	
			24 h	48 h	24 h	48 h	24 h	48 h	24 h	48 h
*S. aureus* ATCC 25923	CFU/mL	1.5 × 10^8^	0 × 10^0^	0 × 10^0^	0 × 10^0^	0 × 10^0^	0 × 10^0^	0 × 10^0^	0 × 10^0^	0 × 10^0^
	Log_10_ (CFU/mL)	8.176	0	0	0	0	0	0	0	0
	Log Reduction	-	8.176	8.176	8.176	8.176	8.176	8.176	8.176	8.176
	LR (%)	-	100	100	100	100	100	100	100	100
*E. coli* ATCC 25922	CFU/mL	1.5 × 10^8^	23.3 × 10^2^	11.68 × 10^2^	9.2 × 10^1^	2.2 × 10^1^	8.3 × 10^1^	1 × 10^0^	0 × 10^0^	0 × 10^0^
	Log_10_ (CFU/mL)	8.176	3.367	3.067	1.963	1.342	1.919	0	0	0
	Log Reduction	-	4.809	5.109	6.213	6.834	6.257	6.257	8.176	8.176
	LR (%)	-	99.920	99.999	99.999	99.999	99.999	99.999	100	100

## Data Availability

Data that support the findings of this study are contained within the article.

## Supplementary Materials

The following supporting information can be downloaded at: https://www.mdpi.com/article/10.3390/gels8070439/s1, Figure S1: GC-MSD/FID gas-chromatography diagram of clove essential oil; Figure S2: Diagram of work steps: Time kill assay; Figure S3: Antibacterial assay test of PCurd, PVA, and CEO against S. aureus (a) and E. coli (b); Table S1: Test results of antibacterial activity of PCurd, PVA, and CEO.
