# Supplementary material for: Phosphorylated Curdlan Gel/Polyvinyl Alcohol Electrospun Nanofibres Loaded with Clove Oil with Antibacterial Activity
Source: Gels. 2022 Jul 13;8(7):439. doi: 10.3390/gels8070439 (PMC9319135; doi:10.3390/gels8070439)
Supplement: Supplementary file 1 [file gels-08-00439-s001.zip › gels-1773924-supplementary.pdf]

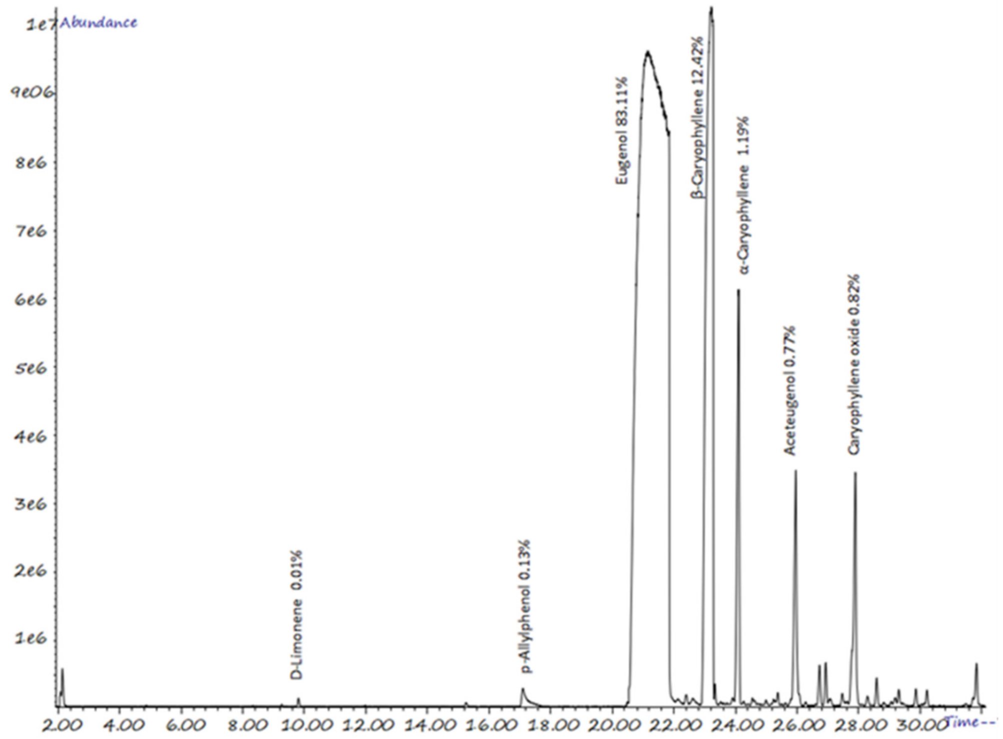

Figure S1. GC-MSD/FID gas-chromatography diagram of clove essential oil

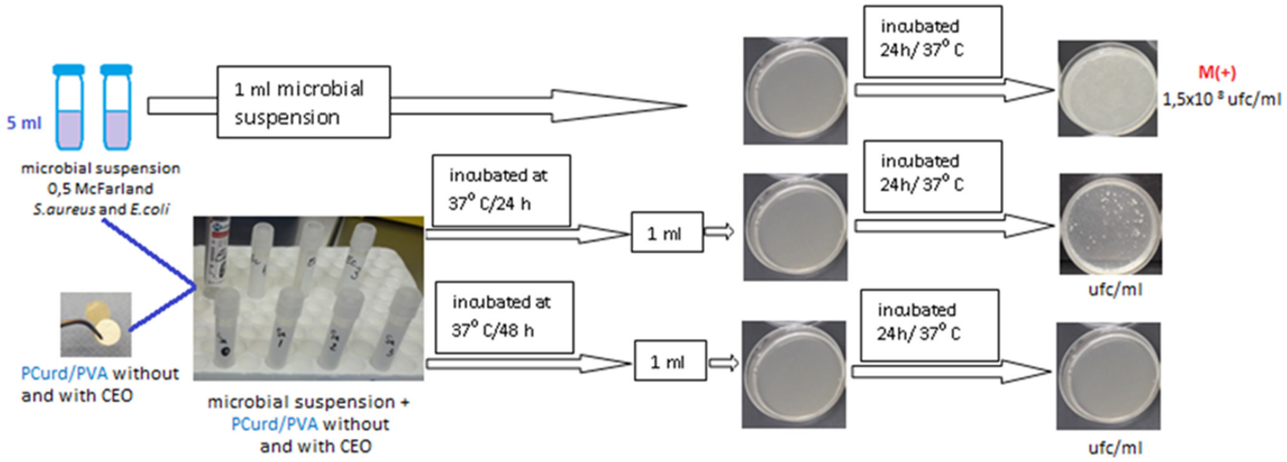

Figure S2. Diagram of work steps: Time kill assay

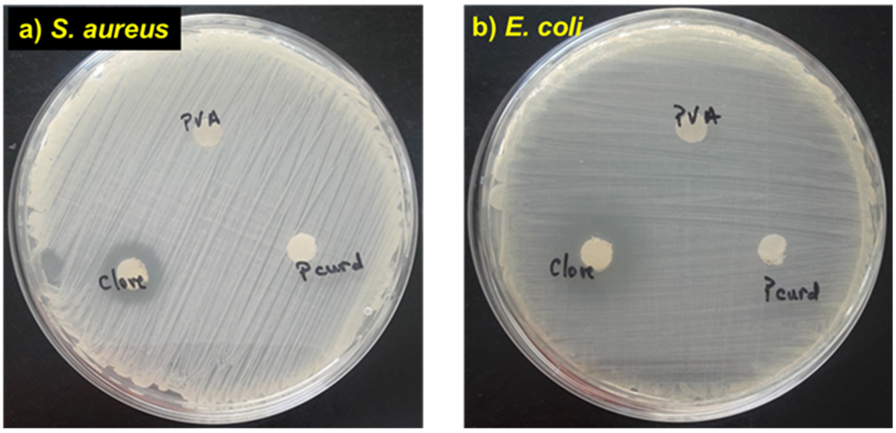

Figure S3. Antibacterial assay test of PCurd, PVA and CEO against S. aureus (a) and E. coli (b)

Table S1. Test results of antimicrobial activity of PVA, PCurd and Clove essential oil

Sample cod	*S. aureus* ATCC 25923 [mm]	*E. coli* ATCC 25922 [mm]
PVA	-	-
PCurd)	-	-
CEO	14	9,94
